# Synthesis of UV-11 MOF and Its Characterization by Cyclic Voltammetry

**DOI:** 10.3389/fchem.2020.00617

**Published:** 2020-08-04

**Authors:** Ingrid Guadalupe Meza-Pardo, Alfredo A. Morales-Tapia, María Aurora Veloz Rodríguez, Victor Esteban Reyes-Cruz, Miguel Perez-Labra, Gustavo Urbano-Reyes, José María Rivera-Villanueva, Jose Angel Cobos-Murcia

**Affiliations:** ^1^Laboratory Electrochemistry Process, Institute of Basic Sciences and Engineering, Academic Area of Earth Sciences and Materials, Autonomous University of the State of Hidalgo, Pachuca, Mexico; ^2^LADISER Organic Chemistry, Faculty of Chemical Sciences, Universidad Veracruzana, Orizaba, Mexico

**Keywords:** characterization-analysis, electroanalysis, MOF (Metal–Organic Framework), interface mechanical behavior, micro supercapacitor, electrochemistry (cyclic voltametry)

## Abstract

In this work a Metal–Organic Framework (MOF) was prepared using a solvothermal method, taking as precursors 1. 2-di-(4-pyridyl)-ethylene, 1.2.4.5-benzenetetracarboxylic acid and Co(No_3_)_2_-6H_2_O. This MOF was called UV-11 and was evaluated using microscopic, spectroscopic and electrochemical techniques. According to the obtained results, the melting point of the compound is located in a higher interval than its precursors. Stereoscopic microscopy analysis shows the presence of pink crystals in the form of needles. MEB technique displays a laminar morphology as well as crystals with approximate sizes (36 mm wide and 150 mm long). EDS analysis corroborated the presence of precursor elements such as cobalt, carbon and oxygen. Furthermore, the XRD technique shows the cobalt-related phases in the sample, which is cobalt bis (pyridine-6-carboxylic-2-carboxylate). A modified carbon paste electrode was prepared using MOF UV-11 and by cyclic voltammetry electrochemical technique, semi-reversible redox processes are identified, as well as thermodynamic and kinetic parameters were obtained with the Laviron equation, and electrochemical performance properties from the cyclic voltammetry experimental data.

## Introduction

Since ancient times, a variety of porous materials have been used, for instance: cell membranes, porous polymers, and some natural materials such as rocks, foams and soils (clays), which are classified as low- or medium-porosity materials (Zdravkov et al., 2007). Nowadays, there are materials with excellent microstructural characteristics (high porosity), such as MOF (metal–organic frameworks); which are crystalline materials with high porosity, belonging to hybrid-material classification, due to the presence of chemical bonds between metal and ligand ions (Zhou et al., 2012), constituting coordination links and the ability to grow in different dimensions such as 2D and 3D (Wang et al., 2005).

MOF have been of great interest due to the wide amount of applications, for instance, catalysis (Shen et al., 2016), magnetism (Tian et al., 2014), luminescence (Allendorf et al., 2009), gas detection (Drobek et al., 2016), water purification (Ma et al., 2017), gas storage (Li and Yang, 2007), adsorption properties (Kaur et al., 2019), and supercapacitors (Lee et al., 2009). The latter is another great advantage of MOF, since the structural flexibility vs. external stimuli (changes in temperature and pressure), that varies their porosity. For this reason, it is possible to promote their use as molecular sieves (Bux et al., 2010) and drug-releasing capsules (Orellana-Tavra et al., 2016), among other things. This versatility in their application is due to the properties such as the high specific surface area of >1,000 m2/g, a regular and adjustable pore texture, flexibility in surface chemistry, and high chemical and thermal stability (Xia, 2011).

MOF are porous structures that can also be called Porous Coordination Polymers (PCPs), Microporous Coordination Polymers (MCPs) or Porous Coordination Networks (PCNs). These materials are known as hybrids because their structure is composed of inorganic nodes and organic linkers through covalent coordination (Vogel, 2012), which allows for different MOF to be designed.

Various techniques are used in their synthesis, notably mechanochemistry (Klimakow et al., 2010), electrochemical (Van Assche et al., 2012), sonochemistry (Son et al., 2008), microwave (Topologies et al., 2012) and solvothermal techniques (Mulyati et al., 2015). The main advantages these techniques promote are the high crystallinity, short-time reaction, and larger crystal size (Li et al., 2009).

On the other hand, the metallic ion used in MOF synthesis has evolved over time and crystals have been made with different metals such as Cu, Rh, Ru, Mo, Cr, Fe, W, Zn, Cu, Mn, Zr, and Co. However, according to the Cambridge Structural Database (CSD), cobalt is the least used element (1%) in the synthesis of MOF (Wang, 2006). However, cobalt in the metallic center of the MOF could contribute with different properties like a high reactivity through the oxygen interaction with the ligands (Betteridge, 1980), allowing many possibilities of application, such as in magnetic and supercapacitors field. This manner, synthesis of MOF with cobalt represents a theme of great interest in the scientific community.

Thus, to synthesize and characterize MOF with cobalt is the main objective of this work. It was synthesized and reported by our work group in October 2015 (Alfonso-Herrera et al., 2018) in the Cambridge Crystallographic Data Center (CCDC) under the number 1434259, called IUPAC catena-[bis(μ-benzene-1,2,4,5-tetracarboxylate)-tris(μ-4-(2-(pyridin-4-yl)vinyl)pyridine]-tetra-cobalt N,N-dimethylformamide solvate (here called MOF UV-11). However, a full characterization for this MOF has not been reported, in spite of it has already been used by other research groups in the post synthesis of this compound for photocatalytic applications in hydrogen evolution degradation of indigo carmine dye (Alfonso-Herrera et al., 2018).

In this work the MOF UV-11 synthesis through a solvothermal technique using cobalt metal ions was carried out, as well as the analytical characterization using electrochemical voltammetry to elude the pathways of the redox electron transfer process, while the faradaic reaction occurs. In particular, because this type of reaction is used to design and build sensors. So, the voltammetry is useful in elucidating mechanisms and estimate thermodynamic, kinetic and electrochemical performance properties.

## Experimental

### Synthesis of MOF UV-11

For MOF UV-11 synthesis, all the used analytical grade reagents were provided by Sigma-Aldrich. The precursors were weighed in a RADWAG XA220 analytical balance, firstly, 0.2743 mmol of 1,2-di-(4-pyridyl)-ethylene, 0.5486 mmol of Co(NO_3_)_2_-6H_2_O, and 0.2743 mmol of 1,2,4,5- benzenetetracarboxylic; subsequently, they were dissolved in 5 ml of Dimethyl Formamide (DMF). In addition, the resulting solution was placed in stainless steel-coated Teflon reactors, being heated inside of a muffle furnace at 90°C for 72 h. Then, all of the reactors were cooled to room temperature and a filtering process was carried out, whereby obtaining the MOF crystals. Finally, MOF UV-11 activation was performed in agitation for 30 min in ethanol. The activation of the MOF is necessary so that it can have a permanent pore size thus avoiding host molecules, this without affecting the structural capacity (Mondloch et al., 2013). Crystals obtained this manner are higher than that of the mechanical synthesis.

### Characterization

Melting point characterization was conducted using Electrothermal fusiometer IA-9200 model 10034137/01. The Z potential of MOF UV-11 was carried out with Zetasizer Nano series from Malvern Instruments. PH solutions were prepared with different values (4, 5, 6 and 7) using an approximate concentration of 185 ppm of MOF UV-11. Stereoscopic microscopy technique was performed using a Motic stereo microscope trinocular model bA310 Pol. For microscopic characterization and energy dispersive spectroscopy (MEB/EDS), JEOL model IT-JSM-300 equipment was utilized with an Oxford X-MaxN model 51-XMX1181 detector. X-ray diffraction was obtained using an INEL EQUINOX 2000 model with a [CoK_α_ = 1.78901 Å] wavelength, Diffraction spectra were obtained in a range of 10–50° for 2θ, with an incremental step size of 0.02°. The data acquisition time constituted 2 s. To make a comparison of the experimental diffractogram, a theoretical simulated model with Mercury 4.3.1 software, was used.

### Preparation of Britton–Robinson Buffer Electrolyte

For the preparation of the supporting electrolyte, a buffer solution called Britton–Robinson was used, which is also known as “universal buffer,” due to the wide pH damping range between 2 and 12. Acid H_3_BO_4_ 0.01 M, phosphoric acid H_3_PO_4_ 0.01 M, and acetic acid CH_3_COOH 0.01 M were utilized at a ratio of 1:1:1, respectively; all of which were of the J.T. Baker brand with 99.5% purity. The pH dissolution was adjusted to 7 with a solution comprising sodium hydroxide 0.01 M; this solution was made using Reasol brand reagent 0.01 M with 97% purity. NaCl 0.001 M was added to the obtained supporting electrolyte, this solution was made using Sigma-Aldrich with 99% purity and deionized water.

### Electrode Preparation

The carbon paste electrode represents one of the most convenient materials for modified electrodes preparation, due to their high homogenization capacity, their easy preparation and modification by different methods (Kalcher, 1990; Taylor et al., 2010). The Carbon Paste Electrodes (CPEs) were prepared using graphite powder with a particle size of <20 microns and Nujol oil as a binder, both of which are produced by Sigma-Aldrich. A 2:1 ratio of graphite and binder was utilized. For the preparation of Modified Carbon Paste Electrodes (MCPEs), the same proportion as the CPEs was used, but 3%_w/w_ of MOF UV-11 was incorporated.

### Voltammetry Characterization

Electrochemical analysis was conducted on an Autolab PGSTAT30 Potentiostat-Galvanostat with NOVA 2.0 software, using a typical three-electrode array. A saturated calomel electrode was used as reference electrode, a platinum bar as counter electrode, and CPEs and MCPEs as the working electrodes. Cyclic voltammetry scan started with a zero current potential of E_i_ = 0 and in an anodic direction, at three different potential scanning rates: 25, 50, and 100 mVs^−1^. In addition, the inversion potential evaluation was undertaken from 750 mV (every 50 mV) until reaching the maximum limit of 1,200 mV.

### Thermodynamic, Kinetic and Electrochemical Performance Parameters

For thermodynamic and kinetic parameters determination of the modified electrode, it was used experimentally adjusting data to the mathematical parameterised model, according to the Laviron equation (Huang et al., 2010) with the Levenberg–Marquardt iteration, in Origin v. 9.1 software.

(1) $$\begin{array}{l} E_{p}=E^{0}+\frac{2.303RT}{\alpha nF}\left[log\;\left(\frac{RT}{\alpha nF}\left(k_{s}\right)\right)\;-log\;\left(v\right)\;\right] \end{array}$$

(2) $$\begin{array}{l} E_{p}=A+B\left[C\;-log\;\left(v\right)\;\right] \end{array}$$

(3) $$\begin{array}{l} A=E^{0} \end{array}$$

(4) $$\begin{array}{l} B=\frac{2.303RT}{\alpha nF} \end{array}$$

(5) $$\begin{array}{l} C=\left[log\;\left(\frac{RT}{\alpha nF}\left(k_{s}\right)\right)\;-log\;\left(v\right)\;\right] \end{array}$$

(6) $$\begin{array}{l} ip=\frac{nFQv}{4R\Gamma } \end{array}$$

Where: α is the electron transfer coefficient, *Ks* is the standard velocity constant of the reaction surface, ν is the potential scan rate, *n* is the electron transfer number, *F* is the Faraday constant, and *E*_0_ is the formal potential.

The electrochemical assessment of the modified electrode from voltammetry data was performed in a potential range from *E*_*OCP*_ to 0.7 V and was calculated using the Equation (1); where, CA is the specific areal capacitance, ν is the scan rate, *E*_*f*_ y *E*_*i*_ are the final and initial potential of discharge, *j* is the current density and *E* the potential. On the other hand, the Ragone graph was drawn to show and compare results of EMPC against EPC and evaluate the electrochemical performance of the modified electrode. The specific areal energy density (*E*_*A*_) and the specific areal power density (*P*_*A*_), was calculated using the Equations (8) and (9) respectively (Yang et al., 2017).

(7) $$\begin{array}{l} C_{A}=\frac{1}{v\left(E_{f}-E_{i}\right)}\int_{E_{i}}^{E_{f}}jdE \end{array}$$

(8) $$\begin{array}{l} E_{A}=\frac{C_{A}^{*}\left(E_{f}-E_{i}\right)}{2} \end{array}$$

(9) $$\begin{array}{l} P_{A}=\frac{E_{A}}{\left(E_{f}-E_{i}\right)/v} \end{array}$$

## Results and Discussion

### Characterization of MOF-UV 11

The method used has a synthesis yield of 72% of the MOF UV-11, and previously activation demonstrated to be necessary in order to keep a permanent pore size to avoid host molecules, without affecting the structural capacity (Farha and Hupp, 2010). The synthesized compound presented a melting point of 292–293°C and show a zeta potential of −10.47 ± 0.99, −8.86 ± 0.81, −10.70 ± 0.49 and −14.60 ± 0.46 mV, for pH of 4, 5, 6, and 7 respectively.

**Figure 1:**
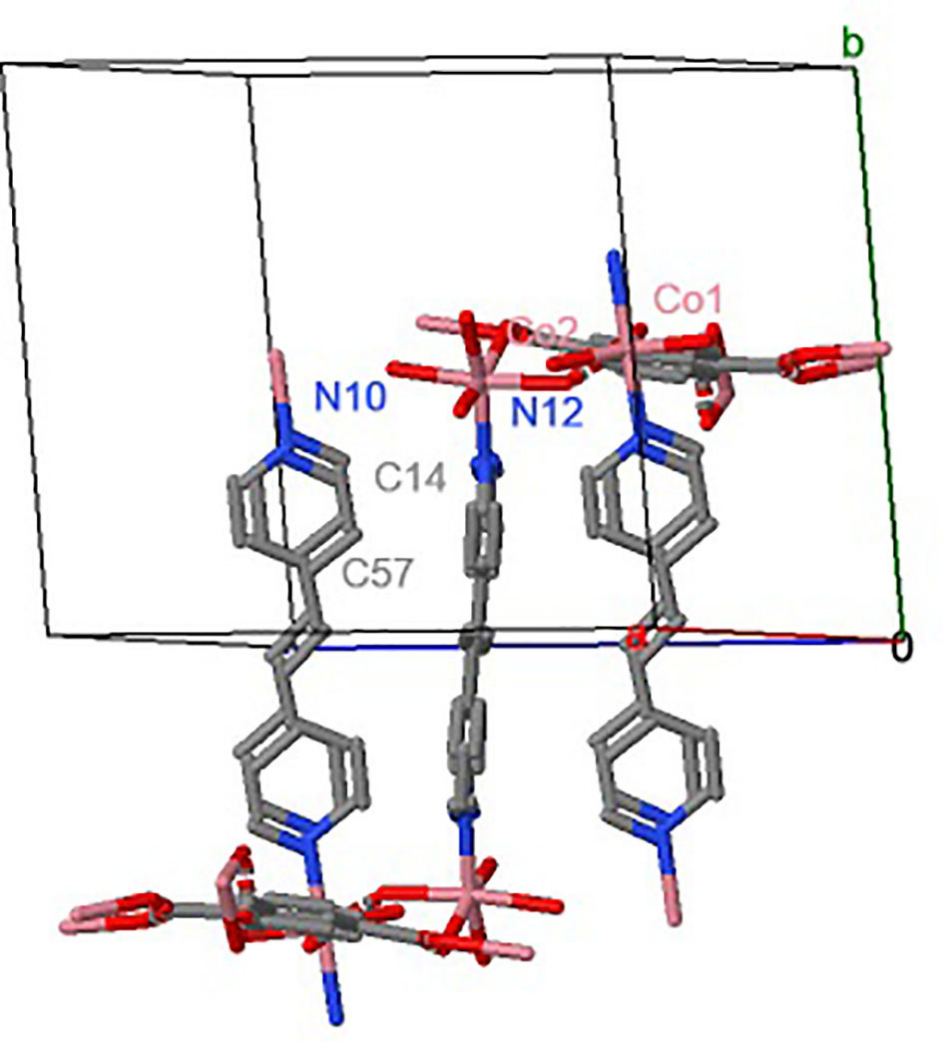
MOF UV-11 unit cell simulation.

Figure 1 shows the MOF UV-11 unit cell simulation, corresponding to the molecule simulated using Mercury 4.3.1 software. In the Figure 1 the hydrogen atoms were omitted for better visualization of the molecule structure. The structure of the compound has two cobalt ions. Firstly, the Co1 ion is coordinated with two nitrogen atoms and three oxygen atoms. Meanwhile, the Co2 ion is coordinated with five oxygen atoms and one nitrogen atom; therefore, the structure and the form of coordination have a triclinic crystalline system and a volume unit cell of 1922 Å.

To corroborate the correct synthesis of the MOF UV-11, a melting point was evaluated, which is different from that of the precursors; being assumed the presence of greater stability in comparison of the precursors, because its high melting point and crystalline structure. On the other hand, the results of Z potential indicate that all over the range of pH evaluated, there is not significant variability, presenting an average of−10 mV; this implies that the MOF is stable in the range evaluated because of the lack of interaction with the electrolyte. This is consistent with the MOF's prime requirements showing structural stability to changes in the pH values (Howarth et al., 2016). These values are attributable to the molecule structure that has functional groups such as carbonyls and aromatic groups, which cause the polar molecule behavior and do not present repulsion toward the molecules in the electrode interface. Also, at this range of pH, the carbonic groups have their acid constant pK_a_ uniform.

**Figure 2:**
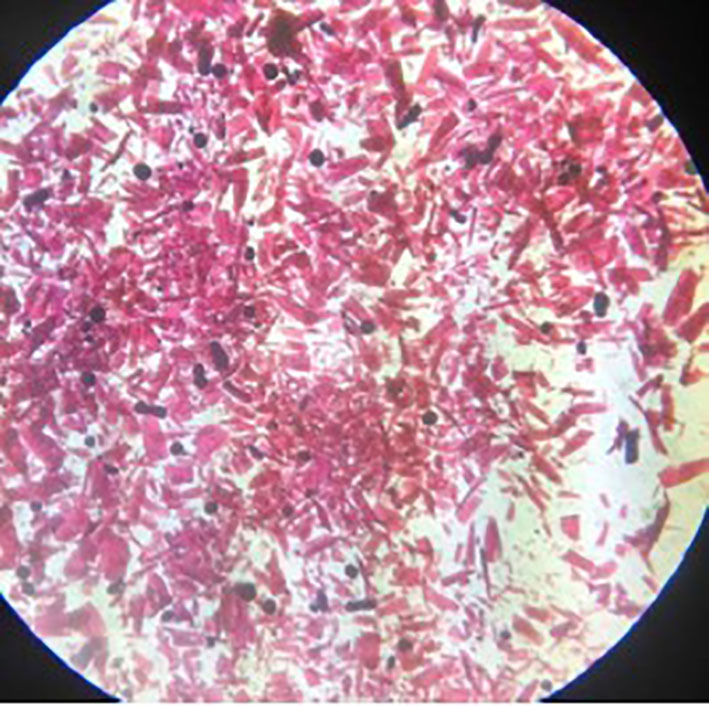
Stereoscopic microscopy of MOF.

Figure 2 shows the stereoscopic micrograph of MOF UV-11 crystals, which have an elongated morphology with pink-violet colouration; this tone is attributed to the presence of cobalt in the metal–organic framework.

The crystals shown have different sizes and thicknesses, attributed to the solvothermal production method. In contrast to other techniques such as the mechanochemical, the smaller crystal dimensions are due to the interaction between mechanical elements and produce results presenting a decrease in magnitudes. The melting point of 1,2-di-(4-pyridyl)-ethylene is in the range of 148–152°C. Moreover, the 1,2,4,5-benzenetetracarboxylic acid is in the range of 281–284°C and Co(NO_3_)2-6H_2_O does not exceed 56°C.

**Figure 3:**
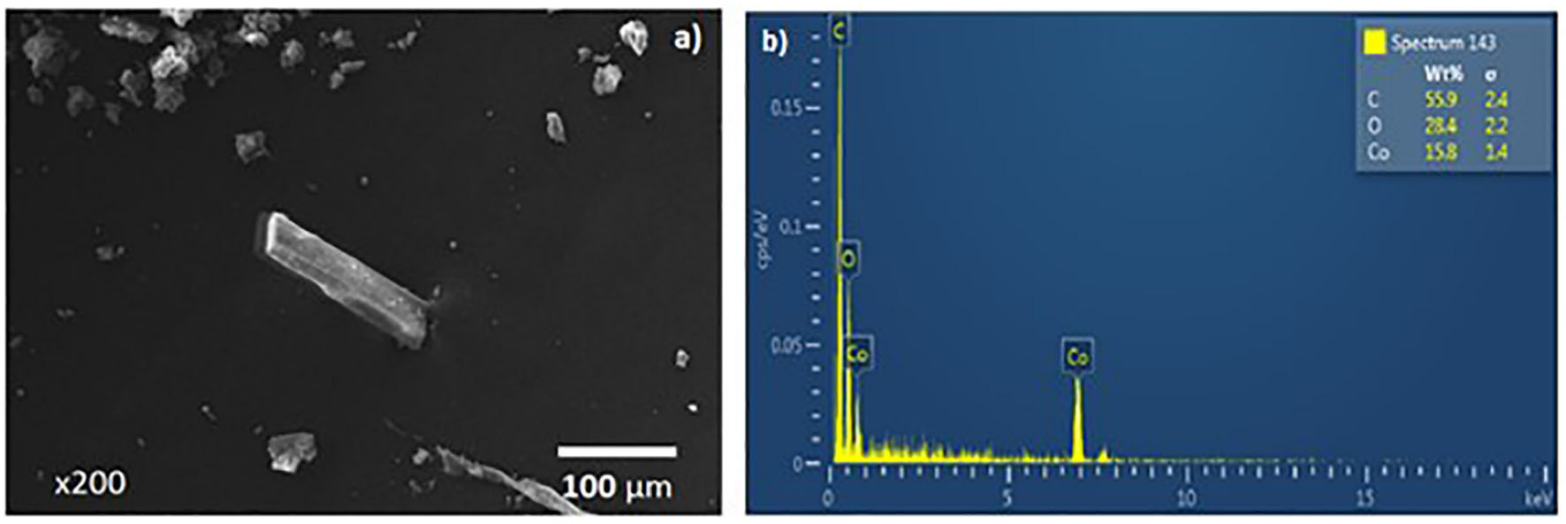
MOF micrograph **(a)** and dispersed electron spectrum **(b)**.

Figure 3 shows micrograph with a 200x magnification at 20 KV (3a) and an EDS analysis of MOF crystals (3b). Figure 3a shows the presence of agglomerates of different sizes fluctuating between 25 and 55 μm.

On the other hand, MOF UV-11 shows microscopic properties such as a rectangular form of the crystal with dimensions of approximately 173 μm in length and 40 μm in width, with a contact surface area of 6932 m^2^. In addition, the EDS analysis corroborated that the MOF crystal (Figure 3b) contains the precursor elements. These components are joined in the metal–organic framework, constituting a mass ratio proportion of 55.9%_w/w_ of carbon, 28.4%_w/w_ of oxygen, and 15.8%_w/w_ of cobalt and according to the simulation shown in Figure 1. The structure and the coordination form have a triclinic crystalline system and an unit cell of 1922 Å volume.

**Figure 4:**
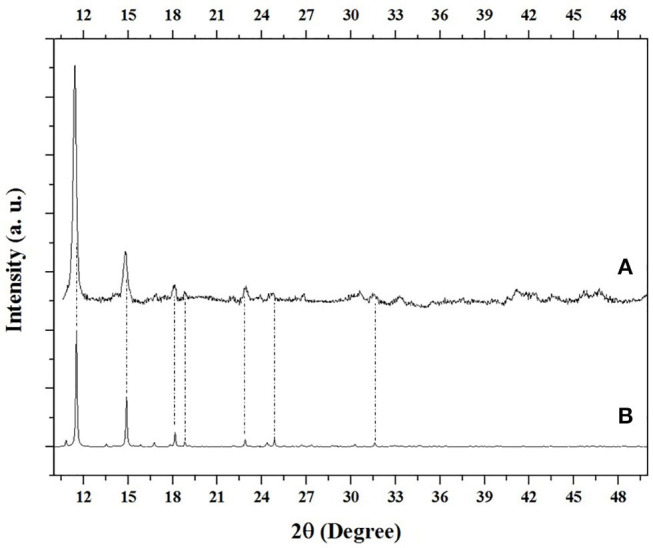
MOF theoretical diffraction comparison **(A)** experimental and **(B)** theoretical.

Figure 4 shows the DRX results, Figure 4A shows MOF UV-11 experimental results. Meanwhile, Figure 4B corresponds to the theoretical diffractogram collected from the Cambridge Crystallographic Data Center (CCDC).

According to XRD analysis, it was found that it is in agreement to the experimental diffractogram (Figure 4A), with the theoretical diffractogram (Figure 4B), respect to 2θ at: 11.51°, 14.91°, 18.15°, 18.80°, 22.87°, 24.86° and 31.58°. Likewise, Herrera et al. in 2018, reported the theoretical diffractogram (Figure 4B), obtaining the same angles reported here. On the other hand, it is possible to corroborate the peaks absence of cobalt nitrate hexahydrate, used as a precursor, according to the PDF-25-1219 card (Glaspell et al., 2007).

### Voltammetry Characterization

**Figure 5:**
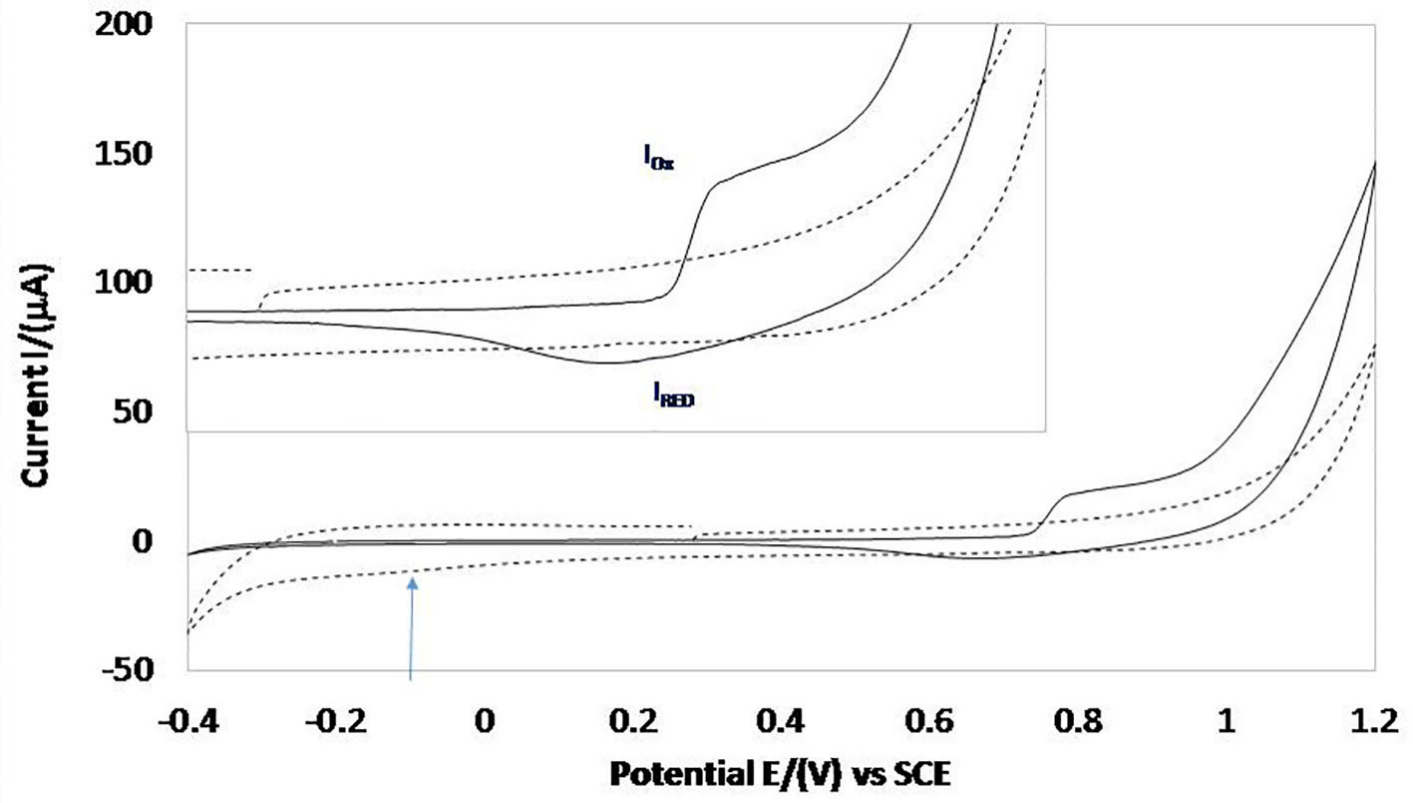
Cyclic voltammetry comparison of MCPE electrode (solid line) and CPE (discontinued line) at a scan rate of 25 mVs^−1^.

Figure 5 shows the cyclic voltammetry comparison of the Carbon Paste Electrode (CPE), that works as a control of the electrochemical response in the absence of the MOF, and the modified carbon paste electrode with MOF UV-11 (MCPE, solid line), with a potential range from −0.4 to 1.2 V at a scan rate of 25 mVs^−1^ and starting in an anodic direction. The inset box shows the redox process expansion that occurs in the range of 0.2–1.1 V.

It is observed that CPE response presents an oxidation process with a higher potential of 0.8 V, which is attributed to the oxygen evolution process. By reversing the scan potential direction, a shoulder is observed at −0.1 V and, subsequently, a reduction process, associated with hydrogen adsorption and evolution, respectively.

On the other hand, the MCPE response shows an oxidation peak (I_OX_) with 0.730 V of potential, attributable to the oxidation of the acid organic linking 1,2,4,5-benzenetetracarboxylic present in MOF UV-11, which matches the oxidation potential range reported in the literature (Talbi et al., 2001) which describes the different modifications in the carboxylic groups induced by electrochemical oxidation, continuing with the potential scan in the anodic-direction process corresponding to the oxygen evolution. Reversing the potential scan to cathodic direction a peak reduction (I_RED_) in 0.670 V can be seen, which is attributed to the carbonyl group reduction of the organic ligand present in the structure of the MOF (RCOO^−^ ↔ RCOO^•^).

In addition, it is important to mention that due to the evaluated potential range, it is not possible to observe the corresponding redox processes of the 1,2-di-(4-piridil) ligand, as these are presented at high reduction potential values (Peroff et al., 2016). This functional group is only an electrical conductor that allows the conduction between the carboxylic and metallic centre groups.

Likewise, the associated processes with oxidation and reduction of Co^2+^ and Co^3+^ are not observed. According to Matheus et al., oxidation is present in a range from 0 to 0.5 V, with a reduction of 0.12 V (Matheus et al., 2008), which is due to the coordination that cobalt presents with the ligands. However, the coordination orbitals of Cobalt allow the storage of the charge received by the carbonyl group. The electron can be stored and subsequently released, by oxidation and reduction of the metal centre that is bound to the ligands.

From results of MCPE it is possible to obtain the corresponding voltammogram through the difference of oxidation peaks (I_OX_) and a reduction (I_RED_) (ΔE = E_C_ – E_A_) with a value of 0.030 V and the analysis using the Nernst equation indicates two electrons with a standard potential reduction of 0.775 V, for the ligand reduction process.

**Figure 6:**
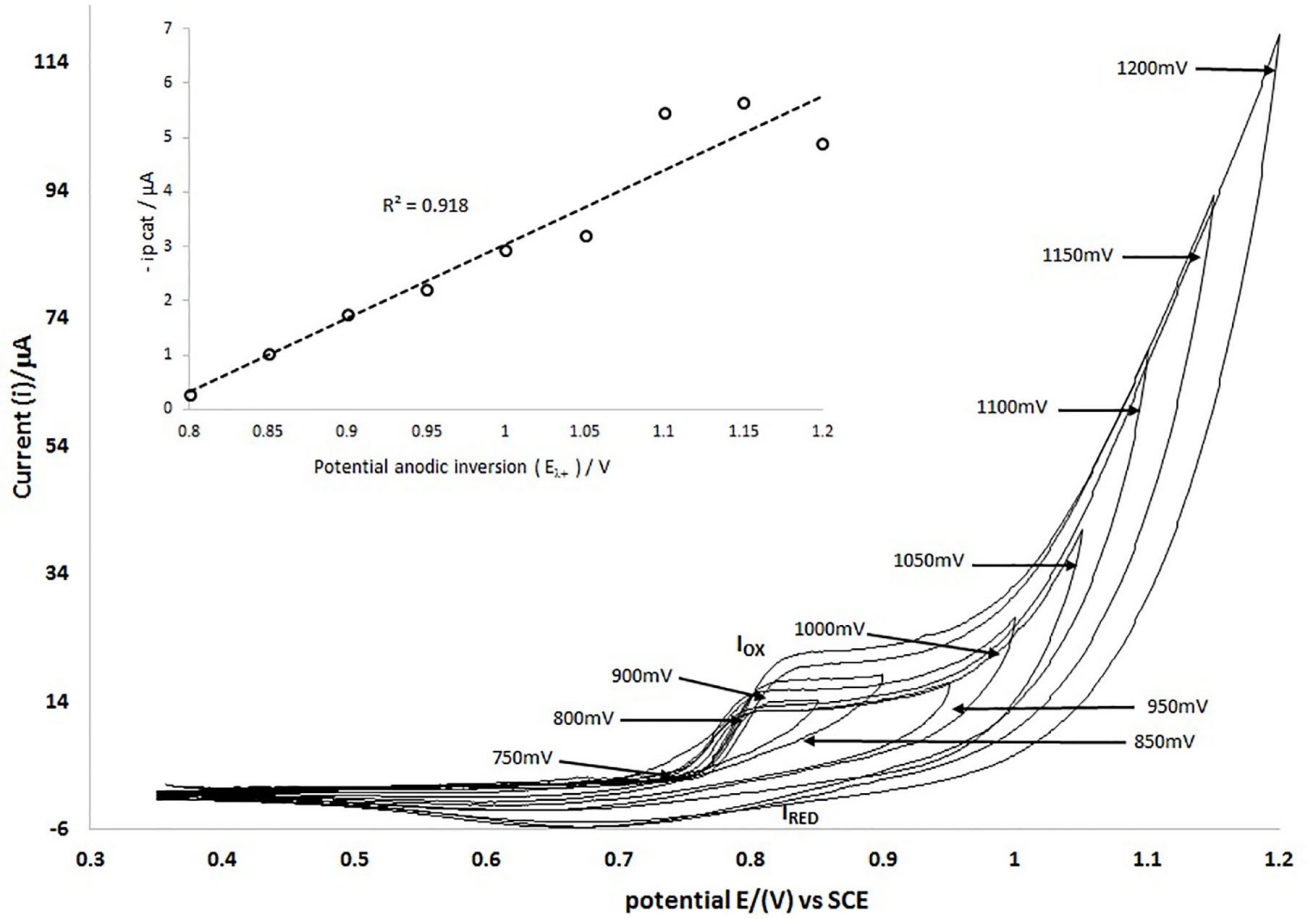
Cyclic voltammetry of MCPE with different positive inversion potentials (E_λ+_) in an electroactive range of 350–1200 mV, evaluating every 50 mV, at a scan rate of 25 mVs^−1^.

Figure 6 shows the cyclic voltammetry comparison at a scanning rate of 25 mVs^−1^ and with different inversion potentials with 50 mV intervals between each and using an electroactive range between 0.350 and 1.2 V.

The results of the investment potential study (Figure 6) indicate that the redox processes correspond to the same chemical species, since both the anodic and cathodic peaks depend on the inversion potential value (see insert), with a correlation coefficient of 0.918. It is important pointing out that voltammetry studies show the stability of the MOF in the modified electrode under the potential conditions evaluated because they show reproducibility and repeatability of the redox process.

**Figure 7:**
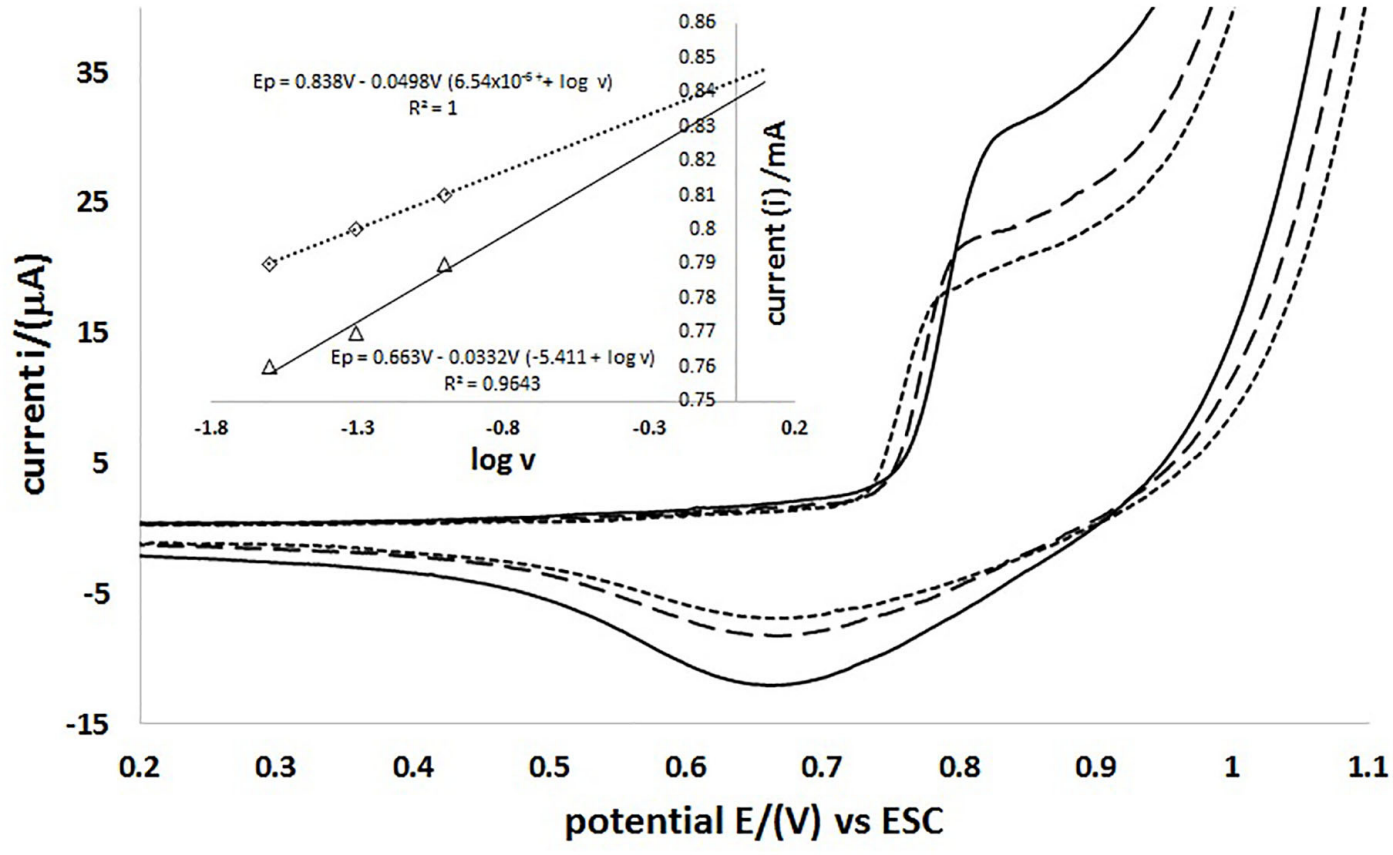
MCPE cyclic voltammetry at different scanning rates vs. SCE 100 mVs^−1^ (–), 50 mVs^−1^ (- - -) and 25 mVs^−1^ (···). The insert shows the current dependency to the logarithm of scan rate.

Figure 7 shows the comparison of cyclic voltammetry at different scan rates (25, 50, and 100 mVs^−1^) using the Modified Carbon Paste Electrode (MCPE), in a potential range from −0.4 to 1.2 V, starting the scan towards anodic direction. It is noticed that there is no significant difference in the anodic capacitive charge when changing the scan rate in the potential range of 0.2–0.7 V and it is also noticeable that the potential of the oxidation peak (I_OX_) begins to happen at the same value. This is because the electroactive species is on the surface of the electrode. It is observed that increasing the scan rate increases the current value of the oxidation (I_OX_) and reduction (I_RED_) peaks. This can be described using the Laviron model (Equation 1), which shows the dependence of potential and current peaks at the scanning rate for processes occurring in confined spaces within an electrode (Thomas et al., 2015).

To obtain the values of the electrochemical parameters the parameterisation of Equation (1) is considered, and broken down in Equation (2); furthermore, each parameter is expressed in Ec. 3–5. Ec. 6 shows the dependence of the current on the scanning rate in terms of the average superficial reaction of the electrode ip. The extension of Figure 7 shows the linear dependence on the value of the anodic peak (I_OX_) and the cathode (I_RED)_ potential value to the scan rate logarithm. The function describes that the behavior of the anodic peak potential is Ep = 0.663–0.033 V (5.411 + log v), with a linear correlation of 0.9643 of the data. Meanwhile, for the potential of the cathode peak at Ep = 0.830–0.0498 V (6.54 × 10^−6^ + log v), there is a linear correlation of 0.9999, which may indicate that there are diffusion mechanisms in the electrode.

With the parameters obtained by the iteration adjustment using the Levenberg–Marquardt algorithm, the thermodynamic and kinetic values of the redox processes described are obtained, as shown in Table 1. With an anodic peak potential (*E*_*pa*_) of 0.624 V and a cathodic peak (*E*_*PC*_) of 0.662 V, there exists a standard redox potential of 0.787 V. This indicates that there is a semi-reversible process, since there is a potential difference (Δ*E*) between the anodic peak and the 0.027 V cathode and the coefficient of the current is 2.706. The standard velocity constant of the reaction surface (ks) obtained was 0.4978 and 0.275 s^−1^ for the anodic and cathodic processes, respectively; with values of the electronic transfer coefficient (α) of the anodic and cathodic processes being 0.47 and 0.79, respectively. The average surface concentration of the electrode reaction was also calculated, constituting 1.012 × 10^−7^ mol^*^cm^−2^, which describes the average of the slope of the maximum peak current vs. the scan rate.

**Table 1:** Thermodynamic values and modeled kinetic parameters using Laviron equation adjustment.

	**E/V=**	**E°/V=**	**ΔE**	**k_**s**_ / s^**−1**^**	**α**	**i_**C**_/i_**A**_**	***Γ*/mol cm^**−2**^**
Anodic	0.624	0.787	0.027	0.4978	0.47	2.706	1.012E-07
Cathodic	0.662			0.2754	0.79		

Therefore, the described processes do not depend on the linear diffusion of the electrolyte to the electrode, but the electroactive species is on the electrode and the process is governed by the rapid transfer species into the same electrode, considering that the higher scanning rate presents a transfer increase of two to three electrons.

On the other hand, the results shown in the voltammetric study indicate the capacitive and resistive behavior on the surface of the modified electrode. It is possible to observe that the current of the capacitive process of the MCPE is lower than that obtained with the CPE. Likewise, the current associated with the hydrogen evolution process in the MCPE is attributable to the electrode conductivity increased and decrease the resistance due to the MOF presence.

**Figure 8:**
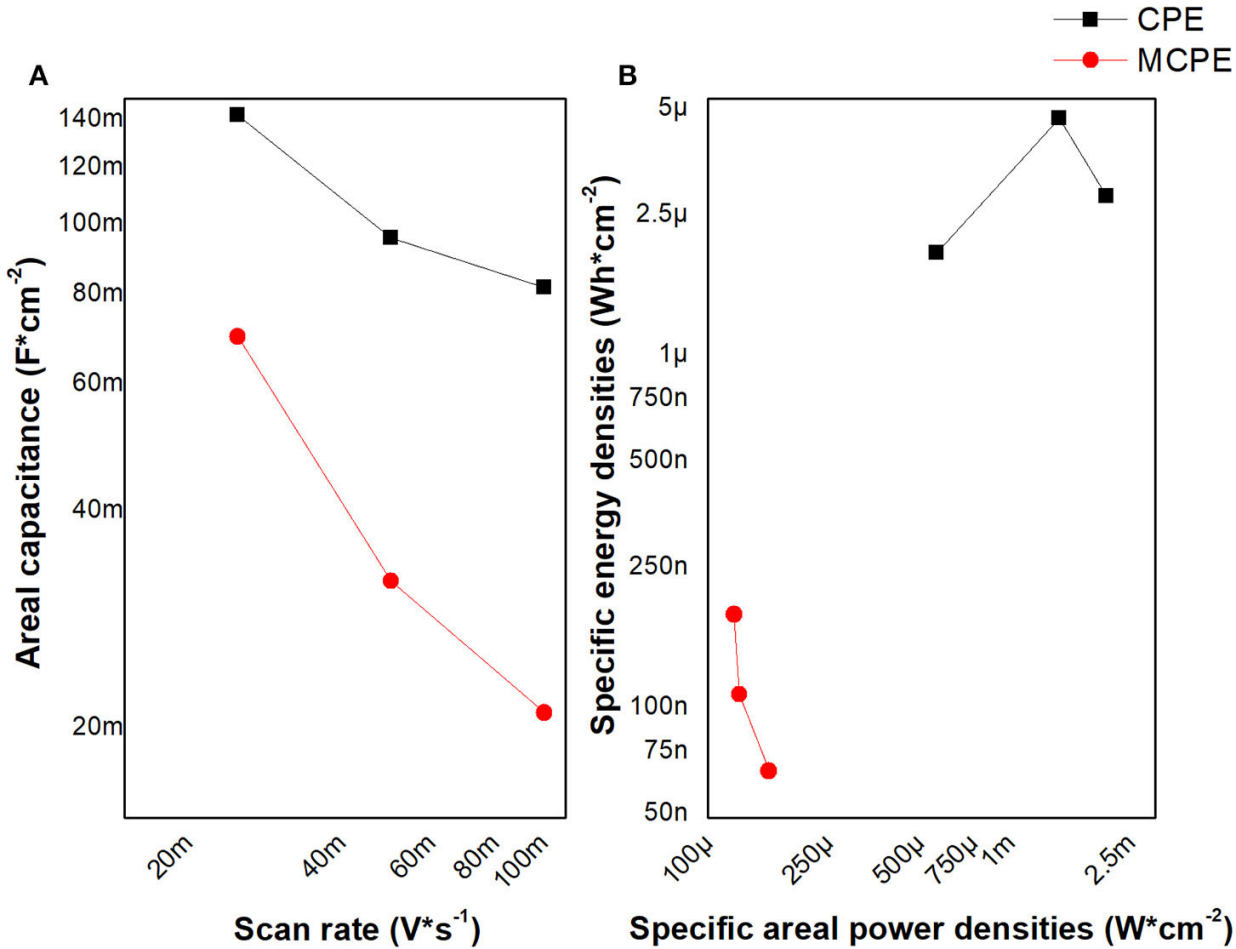
The electrochemical performance of the MCPE using voltammetry data, **(A)** response of the specific areal Capacitance vs. Scan rate and **(B)** the Ragone plot of the electrochemical performance.

Figure 8 shows the comparison of the specific areal capacitance responses of MCPE electrode (solid line) and CPE (discontinued line) to the change of the scan rate (Figure 8A). Figure 8B shows the Ragone plot of the electrochemical performance. The electrochemical performance of the MCPE using voltammetry data, (a) response of the specific areal Capacitance vs. Scan rate and (b) the Ragone plot of the electrochemical performance.

The specific areal capacitance of the MCPE it is lower than the CPE, and both show a diminution of capacitance while the scan rate is increasing. So, its capacitance is lower while increasing the speed of electronic transfer; which favors faradaic processes that can be associated with the electrons in the coordinated orbitals of the metal and pyridyl)-ethylene ligand. These results of CPE are similar with those found for the capacitance and power density on graphite sheet (Mandal et al., 2019). Likewise, their electrodes modified with metal oxides have a capacitance and power density higher than MCPE (Mijangos et al., 2017). The order of energy and power implies that MPCE is an electrochemical microsupercapacitor, which are material useful to miniaturized energy storage systems, like the graphene-based electrochemical microsupercapacitors. These values of energy and power are due to the abundance of active and accessible sites in the interface (Yang and Lu, 2016).

**Figure 9:**
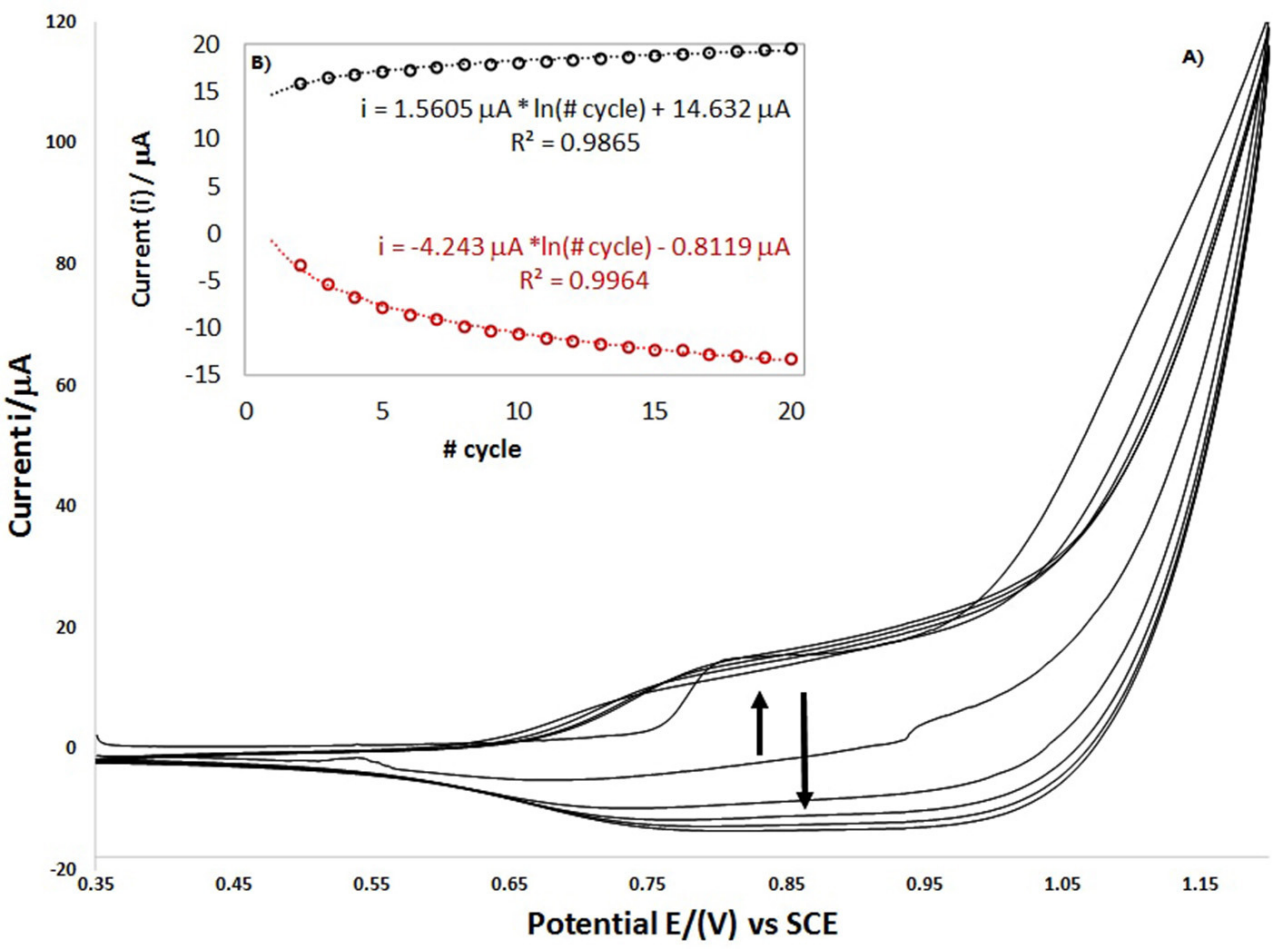
The electrochemical performance of the MCPE using voltammetry data, **(A)** response of the specific areal Capacitance vs. Scan rate and **(B)** the Ragone plot of the electrochemical performance.

Figure 9 shows the cyclic voltammogram repeating the potential scan program 20 times (cycles 1, 5, 10, 15, and 20 are shown). It is observed that the peak current is increased logarithmically according to the number of cycles, with a coefficient correlation of 0.9865 and 0.9964 for anodic and cathodic peaks, respectively. However, from the last three cycles, significant changes are not observed; which may be attributable to MOF crystalline framework activation over the electrode and could be associated with improved conductivity and decreased interface capacitance of the electrode on its surface.

In the Figure 9 it is observed that the peak current is increased logarithmically according to the number of cycles, with a coefficient correlation of 0.9865 and 0.9964 for anodic and cathodic peaks respectively. However, from the last three cycles, significant changes are not observed, which may be attributable to MOF crystalline framework activation over the electrode and could be associated with improved conductivity and decreased interface capacitance of the surface electrode. Likewise, when the program of the voltammetry scanning potential is repeated, the signals are shown in the same potential like the first cycle and do not disappear. In the same way, the current signals grow indicating that the MOF in the modified electrode continues transfer the charge and the redox process.

## Conclusions

The porous coordination polymer obtained using a solvothermal method with a cobalt metal centre (MOF UV-11) presents a melting point in an interval of 292–293°C, which is greater than that of its precursors. Pink crystals (~173 μm in length and 40 μm in width) with a laminar morphology demonstrated the presence of elements such as cobalt, carbon and oxygen. The XRD analysis corroborated the existence of cobalt phase peaks (PDF-00-054-2464) as cobalt bis (pyridine-6-carboxylic-2-carboxylate) supporting the obtaining of MOF UV-11, whose evaluation has not been reported in the literature.

Electrochemical techniques show that MOF UV-11 presents an oxidation peak with a potential of 0.73 V and a reduction peak at 0.67 V, resulting in a semi-reversible process between oxidation and reduction peaks. The voltammetric study at different scanning rates allowed determining thermodynamic values such as the average anodic peak potential at 0.624 V and the cathodic peak at 0.662 V, presenting a formal potential of 0.787 V. There is a difference of potential between the anodic and cathodic peaks of 0.0276 V and the coefficient of the current is 2.706, as well as the kinetic values of the standard speed constant of the anodic process reaction surface of 0.4978 s^−1^ and the cathodic process at 0.275 s^−1^. The electronic transfer coefficient for the anodic and cathodic processes constituted 0.47 and 0.79 values, respectively, and the average concentration surface reaction of the electrode 1.012 × 10-7 mol^*^cm^−2^. These parameters allowed establishing that the process was governed by the electronic transfer of two electrons and the possible electrochemical activation of the MOF structure within the electrode after several voltammetric cycles. The electrochemical performance of the MCPE showed energy and power of an electrochemical micro supercapacitor, useful to miniaturized energy storage systems with a specific areal Capacitance of 40 mFcm^−2^ and a specific areal Energy density of 100 nWh^*^cm^−2^ with a specific areal power density of 120 μW^*^cm^−2^.

## Data Availability Statement

All datasets presented in this study are included in the article.

## Author Contributions

IM-P carried out the organic characterization and synthesis of the materials, as well as the writing of the manuscript. AM-T carried out the organic synthesis and purification of the MOF. MV performed the experimental design and electrochemical tests. VR-C conducted the discussion of the results of the electrochemical characterization. MP-L performed the review and discussion of the results of the characterization by XRD and microscopy. GU-R carried out the design for the preparation of modified electrodes with MOFs. JR-V reviewed and discussed results on MOF synthesis and material characterization. JC-M coordinated the experimental work, discussion of results and writing of the manuscript. All authors contributed to the article and approved the submitted version.

## Conflict of Interest

The authors declare that the research was conducted in the absence of any commercial or financial relationships that could be construed as a potential conflict of interest.
